# Effects of COX-2 inhibition on expression of vascular endothelial growth factor and interleukin-8 in lung cancer cells

**DOI:** 10.1186/1471-2407-8-218

**Published:** 2008-07-31

**Authors:** Yong Ming Zhu, Nor Saadah M Azahri, Danny CW Yu, Penella J Woll

**Affiliations:** 1 Institute for Cancer Studies, School of Medicine and Biomedical Science, University of Sheffield, Beech Hill Road, Sheffield S10 2RX, UK; 2 Cancer Research Centre, Weston Park Hospital, Whitham Road, Sheffield S10 2SJ, UK; 3 Department of Clinical Oncology, School of Medicine and Biomedical Science, University of Sheffield, Sheffield S10 2RX, UK

## Abstract

**Background:**

Cyclooxygenase (COX)-2 has been implicated in tumour progression, angiogenesis and metastasis in non-small cell lung cancer (NSCLC). We speculated that inhibition of COX-2 activity might reduce expression of the pro-angiogenic factors vascular endothelial growth factor (VEGF) and interleukin-8 (IL-8) in lung cancer cells.

**Methods:**

The levels of IL-8, VEGF and prostaglandin E_2 _(PGE_2_) were measured by ELISA. Expression of COX-1 and COX-2 was determined by Western blotting. Inhibition or knockdown of COX-2 was achieved by treating NSCLC cells with specific COX-2 inhibitor NS-398 or COX-2 siRNA, respectively.

**Results:**

We found that NSCLC cell lines produced more IL-8 than VEGF (p < 0.001). In contrast, small cell lung cancer (SCLC) cell lines produced more VEGF than IL-8 (p < 0.001). COX-1 was expressed in all cell lines, but COX-2 was expressed only in NSCLC cell lines. Consistent with this, PGE_2 _was significantly higher in NSCLC cell lines than SCLC cell lines (p < 0.001). We tested these cell lines with a potent specific COX-2 inhibitor NS-398 at concentrations of 0.02, 0.2, 2, 20 μM for 24 or 48 h. The COX-2 activity was reduced in a dose-dependent fashion as shown by reduced PGE_2 _production. VEGF was significantly reduced following the treatment of NS-398 in A549 (by 31%) and MOR/P (by 47%) cells lines which expressing strong COX-2, but not in H460 cell line which expressing very low COX-2. However, IL-8 was not reduced in these cell lines. To confirm these results, we knocked down COX-2 expression with COX-2 siRNA in these cell lines. VEGF was significantly decreased in A549 (by 24%) and in MOR/P (by 53%), but not in H460 whereas IL-8 was not affected in any cell line.

**Conclusion:**

We conclude that NSCLC cells produce much higher levels of IL-8 than SCLC cells whereas both NSCLC and SCLC cells produce similar levels of VEGF. COX-2 is only expressed in NSCLC cells, but not in SCLC cells. VEGF is produced in both NSCLC and SCLC cells regardless of COX-2 expression. However, VEGF production is, at least partly, COX-2 dependent in NSCLC cells expressing COX-2. In contrast, IL-8 production is COX-2 independent in both NSCLC and SCLC cells. We speculate that combined targeting of COX-2 and IL-8 may be useful in the treatment of patients with NSCLC and targeting VEGF may be useful in the treatment of patients with SCLC.

## Background

Lung cancer remains the leading cause of cancer death in many countries worldwide. There is considerable interest in anti-angiogenic drugs as therapeutic agents for lung cancer. Angiogenesis in tumours is promoted through the secretion of a variety of pro-angiogenic factors. Among these, vascular endothelial growth factor (VEGF) is important in many tumour types due to both its potent activity and markedly elevated expression level. Several studies have shown that high levels of VEGF are associated with increased tumour vascularity, advanced stage and poor prognosis in patients with non-small cell (NSCLC) and small cell lung cancer (SCLC) [1-5]. In addition to its angiogenic effects, functional VEGF receptors are expressed on SCLC cells and VEGF induces cell proliferation and migration in these cells [6]. Treatment with a humanized monoclonal antibody to VEGF, bevacizumab (Avastin), prolongs the survival of patients with NSCLC [7].

Interleukin-8 (IL-8), one of the ELR^+ ^CXC family of chemokines, is another potent pro-angiogenic factor and its expression is associated with angiogenesis, tumour progression and survival in patients with NSCLC [8-11]. In addition to its angiogenic effects, IL-8 receptors (CXCR1 and CXCR2) are expressed on lung cancer cells and IL-8 can act as growth/survival factor to these cells [12]. Hence, both VEGF and IL-8 contribute to lung cancer progression through angiogenic and direct mitogenic effects [13,14]. The relative contributions of different angiogenic factors to lung cancer growth are unknown.

Cyclooxygenases (COX) are key enzymes in the conversion of arachidonic acid to prostaglandin (PG) and other eicosanoids including PGE_2_, PGD_2_, PGF_2α_, PGI_2 _and thromboxane A_2_. COX-1 is present in nearly all cells whereas COX-2 is normally undetectable but is inducible under circumstances such as inflammation and cancer. Cancer cells, including NSCLC cells, express high levels of COX-2 protein [15-17]. COX-2 overexpression has been associated with poor prognosis in NSCLC, although a recent meta-analysis challenges this [18]. It is correlated to VEGF and IL-8 expression in NSCLC [19,20]. Selective COX-2 inhibitors have been shown to inhibit the growth and metastasis of several types of cancers [21]. Celecoxib (Celebrex) and rofecoxib (Vioxx) have been tested in clinical trials, but their utilities are limited by cardiac adverse effects. Nevertheless, COX-2 could be a potential target to limit lung cancer growth. However, the mechanism underlying inhibition of angiogenesis and metastasis by targeting COX-2 is not fully understood. The aim of this study was to establish whether there is a direct relationship between COX-2 expression and VEGF and IL-8 production in lung cancer cells.

## Methods

### Lung cancer cell lines, cell culture and reagents

The lung cancer cell lines used were A549, H460, MOR/P (NSCLC) and GLC19, H69, H345, H711, Lu165 (SCLC). All cell lines were grown in RPMI 1640 (Bio Whittaker) with 10% fetal calf serum (Biosera) at 37°C in 5% CO_2_, 95% air. To collect supernatants for VEGF and IL-8 detection, NSCLC (adherent) cells were seeded in 6 well tissue culture plates and SCLC (suspension) cells were seeded in 24 well plates before being treated with various concentrations of the COX-2 inhibitor NS-398 (Cayman Chemical and Calbiochem) in triplicate for 24 h or 48 h. Anti-COX-1 and anti-COX-2 antibodies were purchased from Cayman Chemical. Anti-actin antibody was purchased from Sigma. Antibodies to VEGF and IL-8 for ELISA were purchased from R&D systems. PGE_2 _enzyme immunoassay kit was purchased from R&D systems. The siRNA-COX-2 and siRNA-control were purchased from Dharmacon.

### Enzyme-linked immunosorbent assay (ELISA)

Total VEGF and IL-8 concentrations were determined by ELISA kits as previously described for IL-8 [12]. PGE_2 _was measured using a highly sensitive PGE_2 _competitive ELISA kit according to the manufacturer's instruction. The intensity of colour developed was measured using a Dynatech MR5000 microplate reader at 450 nm optical density (OD) with correction at 570 nm.

### Western blotting

Whole cell lysates were prepared by resuspending cell pellets in CelLytic™M lysis buffer (Sigma) and incubating on ice for 15 minutes before centrifuging at 13,000 rpm for 15 minutes. Protein concentrations were measured in triplicate with the quick start Bradford reagent (Bio-Rad), and 20 μg of protein was added to loading buffer (62.5 mM Tris.HCl pH 6.8, 2% SDS, 10% glycerol, 50 mM DTT and 0.01% bromophenol blue), boiled, and electrophoresed on a 12% polyacrylamide/SDS gel, before being transferred to a Hybond P membrane (Amersham Biosciences). Membranes were incubated in primary anti-COX-2 (1:1000 dilution) or anti-COX-1 antibody (1:200 dilution) for 2 h at room temperature with gentle agitation, and in peroxidase-conjugated rabbit anti-mouse IgG for 2 hour at room temperature before being treated with the ECL detection system and exposed to hyperfilm ECL film (Amersham Biosciences). In some experiments, the membranes were washed and re-probed with anti-actin antibody as control of equal loading.

### Cell growth assay

Cell growth was determined by direct cell counting under the microscope after cells were treated with various concentration of NS-398 for 24 or 48 h. The cells were trypsinized and resuspended in PBS. 50 μl of cell suspension was added to 50 μl of 0.4% Trypan Blue solution, mixed thoroughly and left for 10 min. Viable cells and dead cells (staining blue) were counted in four 1 mm corner squares of a hemocytometer slide. Total cells = (average count/square) × (dilution factor) × 10^4 ^× (predilution volume).

### siRNA transfection

On-target COX-2 plus SMARTpool (COX-2 siRNA) and SiCONTROL Non-Targeting siRNA#1 (control siRNA) were purchased from Dharmacon. The sequences of On-target COX-2 plus SMARTpool (COX-2 siRNA) [GenBank: NM_000963] were GGACUUAUGGGUAAUGUUAUU (duplex 6); GAUAAUUGAUGGAGAGAUGUU (duplex 7); GUGAAACUCUGGCUAGACAUU (duplex 8) and CGAAAUGCAAUUAUGAGUUUU (duplex 9). The siRNAs were transfected into the cells in 12-well plate using DharmaFECT1 transfection reagent according to manufacturer's instructions. Four hours after transfection, the media was replaced by fresh media with 10% FCS. After further 48 h, the media were collected for ELISA and cells were collected for protein extraction.

### Statistical analysis

The results were expressed as mean ± standard error of the mean (SEM). Statistical significance was obtained using unpaired student's t-test. Each experiment was repeated at least 3 times. Values with p < 0.05 were considered significant.

## Results

### IL-8 and VEGF production in NSCLC and SCLC cell lines

**Figure 1:**
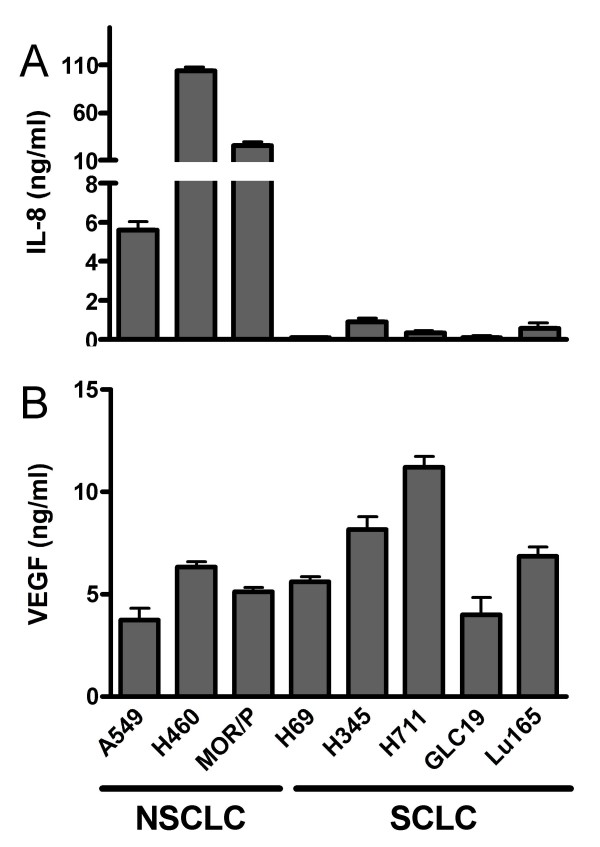
**IL-8 and VEGF production in lung cancer cell lines.** (A). IL-8 protein was measured by ELISA in 48 h conditioned medium of a panel of NSCLC (A549, H460 and MOR/P) and SCLC (H69, H345, H711, GLC19 and Lu165) cell lines. (B). VEGF production was measured by ELISA in the same medium of these cell lines. Each bar is the mean ± SEM of three determinations from three independent experiments.

IL-8 and VEGF were detected by ELISA in conditioned medium after 48 h incubation in a panel of lung cancer cell lines (Figure 1). In all three tested NSCLC cell lines, IL-8 production was significantly (p < 0.001) higher than VEGF production. In contrast, VEGF production was significantly (p < 0.001) higher than IL-8 production in all five tested SCLC cell lines. NSCLC cell lines consistently produced higher levels of IL-8 (above 5.6 ng/ml/10^6^cells) than SCLC cell lines (under 0.9 ng/ml/10^6^cells). However, there was no significant difference in VEGF levels between NSCLC (ranging from 3.7 to 6.3 ng/ml/10^6^cells) and SCLC (ranging from 4.0 to 11.2 ng/ml/10^6^cells) cell lines.

### COX-1 and COX-2 expression and PGE2 production in lung cancer cell lines

**Figure 2:**
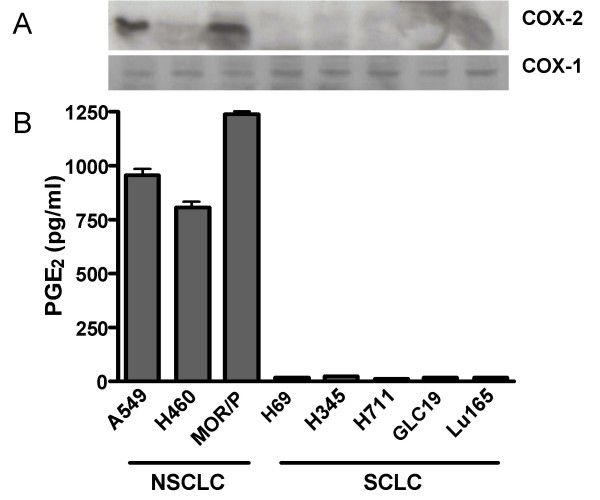
**Expression of COX-1, COX-2 and PGE_2 _in lung cancer cell lines.** (A). Expression of COX-1 and COX-2 by a panel of lung cancer cell lines was determined by Western blotting. 30 μg of total protein was loaded from each sample. These results are representative of three independent experiments (B). PGE_2 _production was measured in 48 h conditioned medium by competitive ELISA. Each bar is the mean ± SEM of three determinations.

Expression of COX-1 and COX-2 by a panel of lung cancer cells was determined by Western blotting. COX-1 was expressed at similar intensity in all NSCLC and SCLC cell lines. In contrast, COX-2 was expressed in all 3 NSCLC cell lines but none of 5 SCLC cell lines. Strong COX-2 expression was seen in MOR/P and A549 cell lines and weak COX-2 was present in H460 cell lines (Figure 2A). The level of PGE_2 _production was measured by competitive ELISA. NSCLC cell lines produced significantly higher levels of PGE_2_(mean 1001 pg/ml/10^6^cells) than SCLC cell lines (mean: 18 pg/ml/10^6^cells, p < 0.001) (Figure 2B).

### Effects of COX-2 inhibition on production of VEGF and IL-8

**Figure 3:**
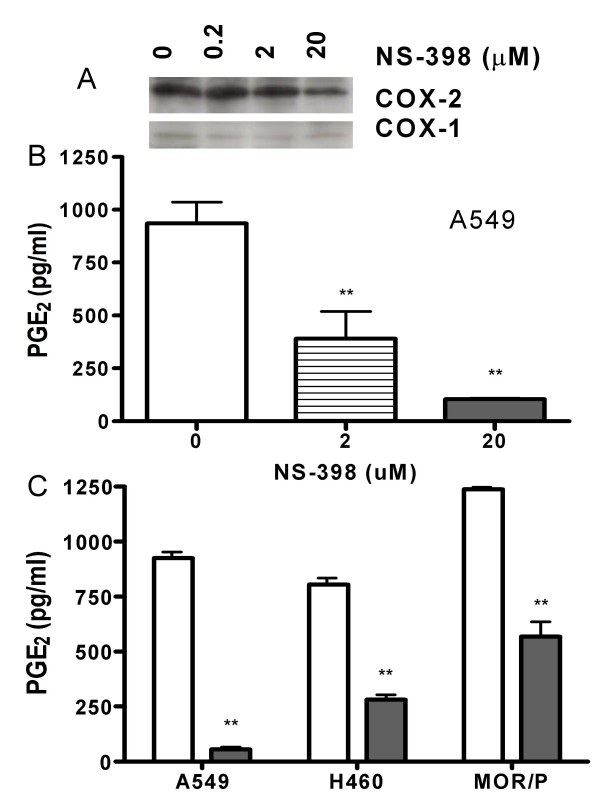
**Effects of NS-398 on expression of COX-1, COX-2 and PGE_2_.** (A). COX-1 and COX-2 expression in MOR/P cells was detected by Western blotting after incubation for 24 h with NS-398 at concentration of 0–20 μM. (B). PGE_2 _production was measured by ELISA in A549 cell line after treatment with NS-398 2 μM (stripe bar) and 20 μM (solid bar) for 48 h, or control (open bar). (C). PGE_2 _production was measured by ELISA in three NSCLC cell lines after treatment with NS-398 20 μM (solid bar) for 24 h, or control (open bar). Each bar is the mean ± SEM of three determinations. ** P < 0.01.

NS-398 is a potent and specific COX-2 inhibitor [22]. To confirm that NS-398 effectively targeted COX-2 in the NSCLC cell lines, we measured PGE_2 _production by ELISA and found that it was reduced in a dose-dependent fashion by NS-398 at 48 h treatment in A549. PGE_2 _was reduced to 390 pg/ml (p < 0.05) by NS-398 at 2 μM and to 105 pg/ml (p < 0.01) by NS-398 at 20 μM from 937 pg/ml, respectively (Figure 3B). At 24 h treatment, PGE_2 _was significantly (p < 0.01) reduced by NS-398 (20 μM) at 24 h in all three tested cell lines (Figure 3C). COX-2 expression was not changed by NS-398 at up to 2 μM, and slightly reduced by NS-398 at 20 μM as shown by Western blotting, whereas COX-1 was not affected (Figure 3A).

**Figure 4:**
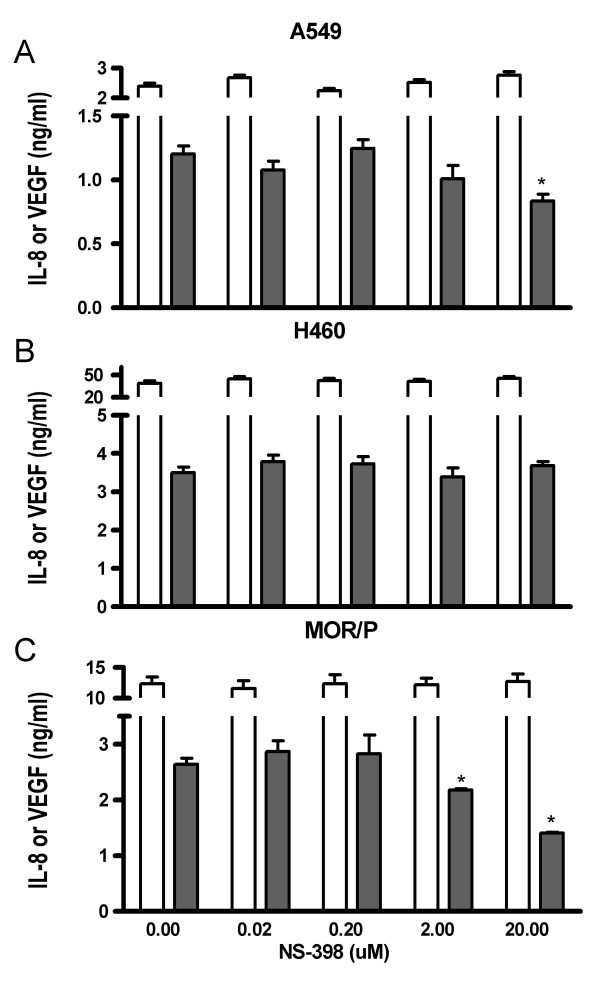
**Effects of COX-2 inhibition on production of VEGF (solid bar) and IL-8 (open bar) in NSCLC cell lines (A) A549, (B) H460 and (C) MOR/P.** Lung cancer cells were treated with various concentrations (0, 0.02, 0.2, 2 and 20 μM) of NS-398 for 48 h. Each bar is the mean ± SEM of three determinations from two independent experiments. * P < 0.05.

To determine whether inhibition of COX-2 affects VEGF and IL-8 production, we treated lung cancer cell lines with various concentrations (0, 0.02, 0.2, 2 and 20 μM) of NS-398 for 24 or 48 h. As expected, NS398 had no effect in SCLC cell lines, which do not express COX-2 (data not shown). However, VEGF production was significantly reduced by NS-398 at 20 μM in A549 (-31%, p < 0.05) and MOR/P (-47%, p < 0.05) cells although VEGF expression was not changed in H460 cells. VEGF was slightly reduced by NS-398 at 2 μM in A549 (-16%, p > 0.05) and MOR/P (-17%, p < 0.05). In contrast, IL-8 production was not reduced by NS-398 in these NSCLC cell lines (Figure 4A, 4B and 4C).

**Figure 5:**
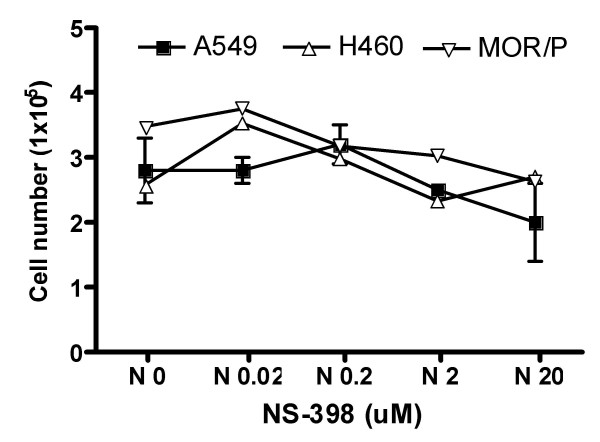
**Effects of COX-2 inhibition on NSCLC cell growth.** Lung cancer cells from three NSCLC cell lines were treated with various concentrations (0, 0.02, 0.2, 2 and 20 μM) of NS-398 for 48 h. Cell numbers were counted by direct cell counting using Trypan Blue stain and the Hemocytometer. Each bar is the mean ± SEM of three determinations from two independent experiments.

To test whether sublethal concentrations of NS-398 used in this study inhibited cell proliferation, we counted cell number using Trypan Blue stain and the Hemocytometer after lung cancer cells were treated with NS-398 for 24 h. There was no significant inhibition of cell growth in any of the 3 NSCLC cell lines at concentrations up to 20 μM of NS-398 (Figure 5).

### Effects of COX-2 knockdown on production of VEGF and IL-8

**Figure 6:**
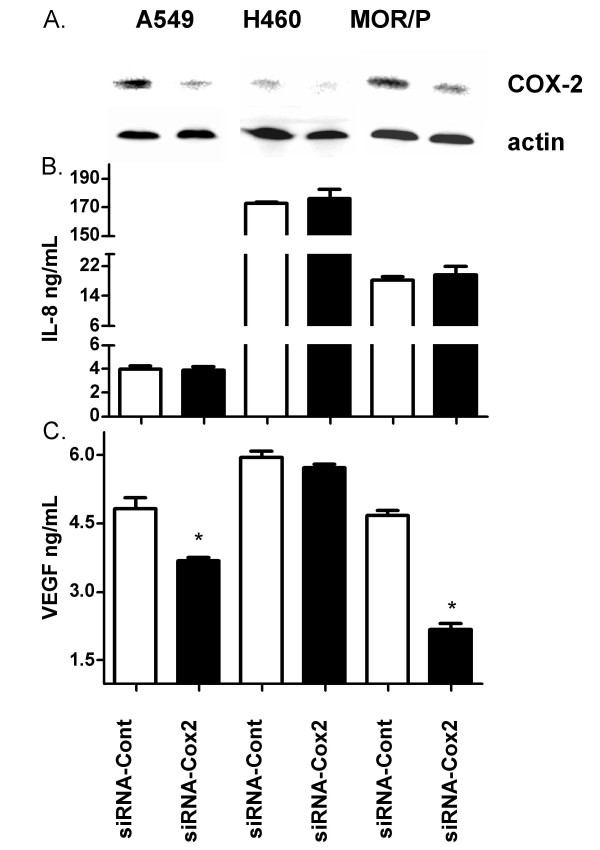
**Effects of COX-2 knockdown on production of VEGF and IL-8 in NSCLC cell lines A549, H460 and MOR/P.** Lung cancer cells were transfected with COX-2 siRNA or control siRNA for 48 h. (A) Expression of COX-2 and actin was detected following transfection for 48 h by Western blotting. (B) IL-8 production was measured by ELISA following transfection of either COX-2 siRNA (solid bar) or control siRNA (open bar). (C) VEGF production was detected by ELISA following transfection of either COX-2 siRNA (solid bar) or control siRNA (open bar). Each bar is the mean ± SEM of three determinations. * P < 0.05.

To further confirm the direct involvement of COX-2 on expression of these factors, we treated NSCLC cell lines with COX-2 siRNA for 48 h. The endogenous COX-2 was markedly knocked down by COX-2 siRNA in all tested cell lines (Figure 6A). Consistent with previous findings with NS-398, IL-8 production was not decreased in any of these tested cell lines (Figure 6B). VEGF was significantly reduced by COX-2 siRNA in A549 (-24%, p < 0.05) and in MOR/P (-53%, p < 0.05), but not in H460 cell line (Figure 6C).

## Discussion

Several studies have shown that COX-2, VEGF and IL-8 are overexpressed in lung cancer compared to normal bronchial epithelium. Most studies have focused on NSCLC since clinical specimens of SCLC are difficult to obtain for research because SCLC patients are rarely operated on. Tas et al [23,24] recently reported that serum VEGF levels were significantly higher in lung cancer patients than healthy controls. Mean serum VEGF levels appeared to be slightly higher in SCLC (1350 pg/ml, range 170 – 3810 pg/ml, n = 34) than NSCLC patients (402 pg/ml, range 121 – 1800 pg/ml, n = 52), but this was not tested statistically.

Here, we have compared and contrasted SCLC and NSCLC using a panel of cell lines. Basal expression of VEGF, IL-8, COX-1, COX-2 and PGE_2 _was determined in three NSCLC and five SCLC cell lines. VEGF was produced at similar levels in all cell lines. NSCLC cell lines produced much more IL-8 than SCLC cell lines. COX-1 was expressed at similar levels in all cell lines, whereas COX-2 was only expressed in NSCLC cell lines, albeit at low levels in H460. Interestingly, high levels of PGE_2 _were not only produced in MOR/P and A549 cell lines as a result of strong COX-2 expression, but also in H460 cell line in which COX-2 expression was weak. This could result from reduced catabolism of PGE_2 _due to low levels of 15-hydroxyprostaglandin dehydrogenase (15-PGDH), a primary enzyme responsible for PGE_2 _metabolism [25]. Previous studies showed that NSCLC cells expressed COX-2 protein [17,26]. However, one study showed that a subset of 20% of SCLC patients (11 out of 54) expressed COX-2 [27]. These data, together with our results and those of Pold et al [20], appear to suggest an association between COX-2 expression and IL-8 production in lung cancer cells. In contrast, VEGF production did not seem to be solely related to COX-2 expression as both COX-2 positive and negative cell lines produced similar levels of VEGF in our study.

COX-2 expression has previously been associated with VEGF in patients with NSCLC [19,20]. Two reports from Dubinett's lab showed that expressions of IL-8 and VEGF were enhanced in NSCLC cell lines transfected with retroviral COX-2 vector [20,28]. The aim of this study was to exam the causal relationship between COX-2 and IL-8 or VEGF in lung cancer cell through inhibition of endogenous COX-2 using either a potent and specific COX-2 inhibitor, NS398 or COX-2 siRNA. NSCLC cell lines were incubated with NS-398 for 24 h and 48 h. NS-398 exerts its effects by directly inhibits COX-2 activity. We demonstrated that COX-2 activity was inhibited by NS-398 as evidence of reduced production of PGE_2 _in these cell lines. We decided to use a maximum concentration of 20 μM in this study because COX-2 inhibition had been demonstrated and cell growth was not significantly inhibited in NSCLC cell lines as shown in Figure 5 at this level. NS-398 has been shown to induce 16% apoptosis in H460 cells at 300 μM in another study [29]. Unexpectedly, IL-8 production was not altered after treatment with NS-398 in these cell lines. However, VEGF was significantly decreased by NS-398 in A549 and MOR/P cell lines, but not in H460. Both of A549 and MOR/P cell lines expressed high levels of COX-2 whereas H460 expressed very low level of COX-2 in our experiments. In some reports, H460 was considered as COX-2-negative cell line [29]. These results suggested that VEGF production was independent of COX-2 in H460 and other COX-2-negative cells such as SCLC cell lines. We further confirm the direct involvement of COX-2 on VEGF production using COX-2 siRNA, and similar results were found. VEGF was significantly reduced in both of A549 and MOR/P, but not in H460. IL-8 was not affected following treatment with COX-2 siRNA in all tested NSCLC cell lines. These results suggest that VEGF is, at least partly, COX-2 dependent in COX-2-expressing NSCLC cells such as A549 and MOR/P. This finding is in agreement with other findings [19,20,28,30]. However, VEGF production is clearly not depend on COX-2 status in other lung cancer cells such as SCLC cells, which expressing no COX-2, and some NSCLC cells including H460, which expressing very low level of COX-2. Our results also suggest that IL-8 is COX-2 independent in lung cancer cells. This finding seems to contradict others [28]. The different findings may be due to the different experiments systems (e.g. to introduce ectopic overexpression of COX-2 or to knock down endogenous COX-2 by siRNA) employed in these studies. Interestingly, Raut et al [31] reported that blocking COX-2 production by NS-398 in pancreatic cancer cell lines did not affect VEGF, bFGF and IL-8 production, but Singh et al [32] found that NS-398 downregulated IL-8 by 30% in a COX-2 transfected MDA-231 breast cancer cell line. These contradictory findings suggest that the relationship between COX-2, VEGF and IL-8 is complex. More studies are required to establish the causal relationship between COX-2 and IL-8 and VEGF in lung cancer. COX-2 inhibitors have been shown to reduce angiogenesis and metastasis in lung cancer [21], our results suggest that anti-angiogenic effects of COX-2 inhibitors might be, at least partly, mediated by inhibition of VEGF, but not IL-8. As IL-8 seems to be independent of COX-2, therefore, there is a strong rationale for combining treatments with inhibitors of COX-2 and IL-8 in NSCLC.

## Conclusion

Our results suggest that NSCLC cells produce much more IL-8 than SCLC cells whereas both NSCLC and SCLC cells produce similar levels of VEGF. COX-2 is only expressed in NSCLC cells, but not in SCLC cells. VEGF can be produced in lung cancer cells regardless of COX-2 expression. However, VEGF production is, at least partly, COX-2 dependent in NSCLC cells expressing COX-2. In contrast, IL-8 production is COX-2 independent in both NSCLC and SCLC cells. We speculate that combined targeting of COX-2 and IL-8 may be useful in the treatment of patients with NSCLC and targeting VEGF may be useful in the treatment of patients with SCLC.

## Competing interests

The authors declare that they have no competing interests.

## Authors' contributions

YMZ designed all experiments, performed some experiments and wrote the manuscript. NSMA and DC–WY performed majority of experiments. PJW contributed to experimental design and manuscript writing. All authors read and approved the final version of the manuscript.

## Pre-publication history

The pre-publication history for this paper can be accessed here:


